# Workflow for health-related and brain data lifecycle

**DOI:** 10.3389/fdgth.2022.1025086

**Published:** 2022-11-30

**Authors:** Petr Brůha, Roman Mouček, Jaromír Salamon, Vítězslav Vacek

**Affiliations:** ^1^ Department of Computer Science and Engineering, Faculty of Applied Sciences, University of West Bohemia, Pilsen, Czech Republic; ^2^ New Technologies for the Information Society, Faculty of Applied Sciences, University of West Bohemia, Pilsen, Czech Republic

**Keywords:** best practices, brain data, data lifecycle, health information system, health-related data, physical data, workflow, ontology

## Abstract

Poor lifestyle leads potentially to chronic diseases and low-grade physical and mental fitness. However, ahead of time, we can measure and analyze multiple aspects of physical and mental health, such as body parameters, health risk factors, degrees of motivation, and the overall willingness to change the current lifestyle. In conjunction with data representing human brain activity, we can obtain and identify human health problems resulting from a long-term lifestyle more precisely and, where appropriate, improve the quality and length of human life. Currently, brain and physical health-related data are not commonly collected and evaluated together. However, doing that is supposed to be an interesting and viable concept, especially when followed by a more detailed definition and description of their whole processing lifecycle. Moreover, when best practices are used to store, annotate, analyze, and evaluate such data collections, the necessary infrastructure development and more intense cooperation among scientific teams and laboratories are facilitated. This approach also improves the reproducibility of experimental work. As a result, large collections of physical and brain health-related data could provide a robust basis for better interpretation of a person’s overall health. This work aims to overview and reflect some best practices used within global communities to ensure the reproducibility of experiments, collected datasets and related workflows. These best practices concern, e.g., data lifecycle models, FAIR principles, and definitions and implementations of terminologies and ontologies. Then, an example of how an automated workflow system could be created to support the collection, annotation, storage, analysis, and publication of findings is shown. The Body in Numbers pilot system, also utilizing software engineering best practices, was developed to implement the concept of such an automated workflow system. It is unique just due to the combination of the processing and evaluation of physical and brain (electrophysiological) data. Its implementation is explored in greater detail, and opportunities to use the gained findings and results throughout various application domains are discussed.

## 1. Introduction

Poor lifestyle leads potentially to chronic diseases and deteriorating physical and mental fitness. To overcome (at least partly) these troubles during aging, prior collection and evaluation of various health-related data accompanied by health interventions for those interested in them could help this unpleasant situation, which mainly affects developed societies. We can measure and analyze multiple aspects of physical and mental health, such as body parameters, health risk factors, degrees of motivation, and the overall willingness to change the current lifestyle in advance. In conjunction with data representing human brain activity, we can obtain and identify human health problems resulting from a long-term lifestyle more precisely and, where appropriate, improve the quality and length of human life.

However, the possibility of interpreting various health-related data and providing subsequent reasonable health interventions means first defining and collecting a large amount of various health-related data that can be processed automatically. It is impossible without using the results of standardization efforts and best practices applied across various domains of health-related data. These efforts and practices significantly impact the entire data collection, storage, processing, and interpretation lifecycle. As a result, these (infrastructure-related) issues need to be addressed, presented, and discussed in the scientific communities so that the experimental work is better reproducible and the data collected can be better analyzed across domains and scales. As technical solutions (technical means to collect, organize, store, annotate, and analyze data) are becoming less of a barrier, and it depends increasingly on knowledge and acceptance of partial solutions, existing standards, and best practices in different domains, this article offers a synthesis of some existing approaches to contribute to the interpretability of collected data and their actual use for communication and possible timely preventive adjustment of the lifestyle of individuals. It is done by providing an overview of some current “standards” and best practices and their integration into a proposed solution.

Health-related data accompanied by metadata are inherently heterogeneous; they are organized and stored in various structures, formats, and data repositories. Related metadata contains various written points ranging from precise data descriptions to only stated basic information based on experimenters’ requirements and task circumstances. Also, metadata can be structured differently and stored in various formats, making the processing or recreating similar experiments somewhat tedious. As a result, retrieving the knowledge from these kinds of data is quite challenging (1). However, it is still no exception that metadata is written down on paper as notes without any used standards.

Recently, the popularity of Cyber-Physical Systems (CPSs) has been on the rise. The thought that wearables, small electronic devices (a fitness armband like FitBit is a good example of this) attached to the surface of the skin, collecting large quantities of medical data (with a sufficient degree of data quality and precision) and enhancing the lifestyle of a person, can be used on a day-to-day basis was adopted by many people. These CPSs can collect large amounts of health-related data. However, each of these CPS devices collects the data in various (generally self-made) data formats, for example, via connection to a Smartphone of the user (2).

Sharing collected data in a thoroughly described fashion is mainly left to the experimenter’s best knowledge; it is up to the experimenter to assess how thoroughly or well-defined the objectives should be. The need to know how thoroughly the collected data should be described for a different party to reproduce the results is often to an open interpretation, which generally leads to different results. Some standards and conventions that apply to health-related data can also be applied to brain data. In our studies and this paper, we focus on the electrical activity of the human brain, i.e., on electroencephalography (EEG) and event-related potential (ERP) data. To bring some widely available standards into this domain, organizations like INCF have proposed how neuroscience data could be collected and stored, so they could be easily accessed and shared across the community (3).

This document emphasizes the best practices regarding the data lifecycle process, i.e., the collection, annotation, analysis, interpretation, and publication of data/results and offers them to a broad scientific audience. Our suggestions will cover the subjects ranging from the original experiment, data collection, storage, and description to processes on how to best store and publish the results. The benefit of a wider audience taking a look at one’s raw data and findings might lead to a healthy debate about the achieved goals (highlighting errors or discovering new findings in the already collected data), as highlighted in (4). This was, for example, emphasized in win-win data sharing in neuroscience (5); there can be a lot of hidden benefits to being discovered when leading a procreative discussion of results. The data need to be stored to be understandable and easy to interpret to make the discussion as frictionless as possible. The general rules of practical data sharing that can be applied to either neuroscientific or physical health data were also mentioned in (6).

Inside the growing field, such as neuroscience, giving such “order” to the collected data is mostly used through the use of a dynamic “ever-evolving” ontology for the current subject (7). These ontologies precisely define the used terms inside the application domain, which again help in easier understanding and reuse of the once-collected data with new research goals. The dynamic ontology will help in this regard that the defined terminology may be used across the scope of multiple subjects and help thus to answer a variety of questions (8). Also, for a truly dynamic ontology, it is necessary to ensure how the changes will be propagated or added in the already existing whole (9). There were already proposed multiple ways how it can be done, for example, through the usage of dynamic web ontology language (dOWL) (10).

In this document, we would like to show and help visualize our best practices focusing on all aspects of creating an experiment and help with the definition and categorization of results/findings through the use of widely used knowledge models (in this specific case, through a dynamic ontology) and publication of the results in an easy-to-understand way. Finally, various research groups worldwide can either discuss these findings or reuse these conclusions for their own specific research without the need to reinvent the wheel.

Since most of these steps seem too abstract, we would like to show a possible way on how such a data lifecycle might look, together with practical examples and underlying data published in widely accessible journals that followed these above-mentioned best practices. In this paper, we will cover the subjects ranging from experiment design, collection of generally heterogeneous data (e.g., heart rate, glucose, body proportions, physical strength with electroencephalography data, and many more), and the description of collected data (for example, by using ontologies) to the publication of both the findings and underlying raw data for a further verification/analysis done by the broad scientific audience.

## 2. Materials and methods

In this chapter, we will focus on showing the current state of the art and the technological background that was utilized during the conceptualization and development of the module architecture utilized by an information system for health-related data collection.

### 2.1. State of the art

The lifecycle (Section 2.1.1) of any entity (such as software or health-related data) should follow key principles (Section 2.1.2). We recognize the functional aspects (processing) and data (objects, subjects) stepping into the process. The descriptions of data and their organizations are various, and we prefer terminologies (Section 2.1.3) and ontologies (Section 2.1.3) when it comes to health-related data. The data properly identified and described are stored in the standardized and interchangeable data format (Section 2.1.4).

#### 2.1.1. Software engineering methodologies and data management lifecycles

The organization of software development and data processing as critical activities to achieve work effectiveness and efficiency has led to defining development methodologies and software/data lifecycles. These methodologies and lifecycles also create a primary platform to achieve another challenge—open, fair, and reproducible science.

**Figure 1:**
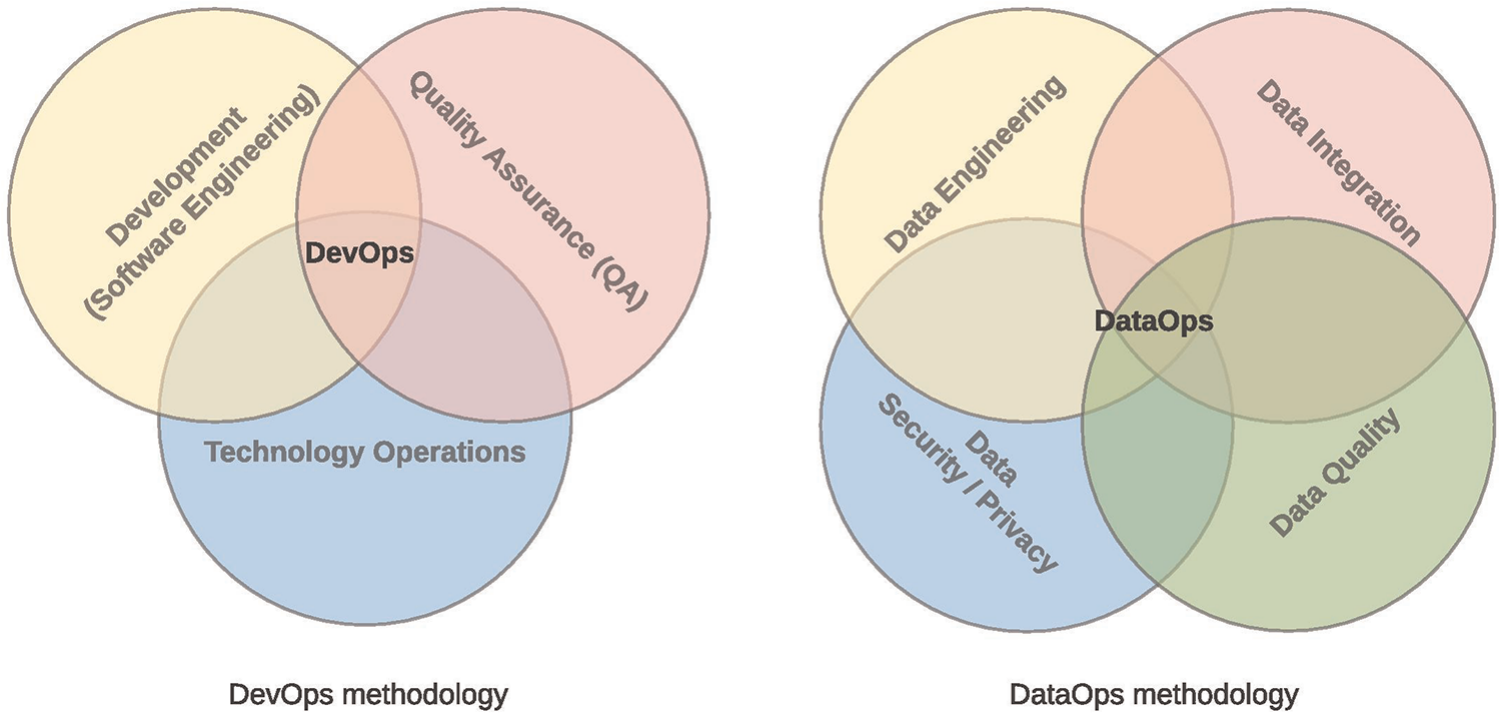
DevOps (16) and DataOps in the enterprise (16).

**Figure 2:**
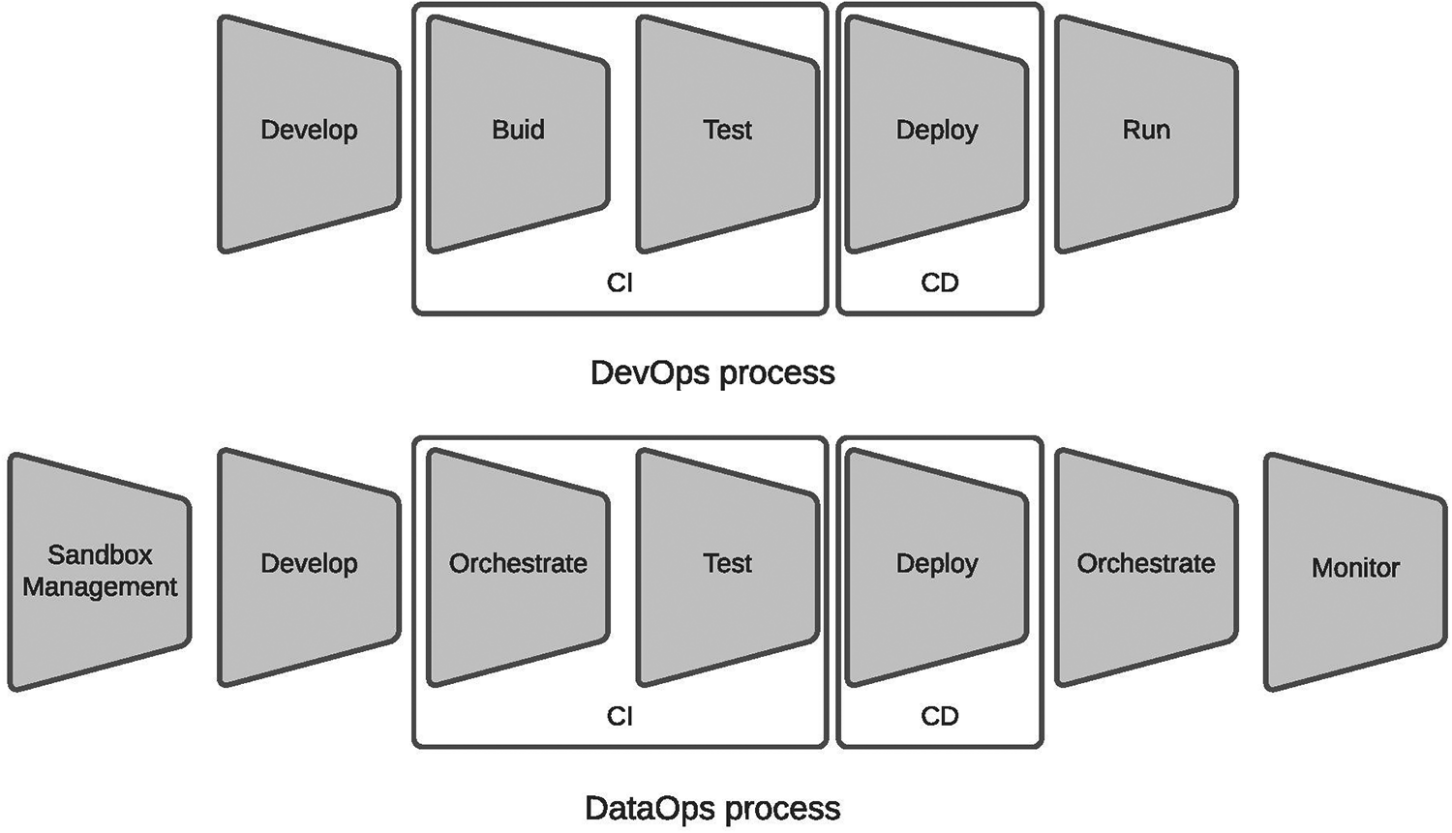
DevOps and DataOps processes (17).

Agile development methodologies have followed waterfall software development methodologies (11). At the same time as the agile methodologies, the era of big data began. It took significant importance in the last decade when cheap data storage and computational power increased exponentially. Agile software development has evolved into a complementary set of practices called DevOps (12–14), where software development (Dev, Software Engineering), IT (technology) operations (Ops), and quality assurance (QA) are present (Figure 1, left part). Processwise, DevOps represents a typical chain for delivering software solutions; it includes software development, building, testing, deployment, and running (Figure 2, upper part).

However, the DevOps does not correspond to the specific needs of big data; thus, DataOps (15) has been introduced. DataOps includes other data-driven disciplines like data engineering, data integration, data security, and data quality (Figure 1, right part). It represents a complete data lifecycle from data preparation and gathering over the transformation to reporting. It brings a bridge between data analytics teams and IT operations. DataOps focuses highly on data pipeline orchestration, data quality, and continuous integration/delivery. It provides the chance to get a consistent and reliable source for data ingestion and reporting and advanced analytics represented by machine learning (ML) models and artificial intelligence (AI) solutions. Next to the processwise qualities, DataOps provides capabilities about data lifecycle, data annotation (relations, the meaning given by ontology, versioning), and data lineage (auditability, explainability).

Processwise, DataOps extends the processing chain to focus more on the data-related products instead of being software-centric. It adds sandbox management for implementing data prototype products, replaces the build process, runs with orchestration, and adds monitoring at the end (Figure 2, lower part).

For machine learning, DataOps moved even further and evolved into the MLOps (18) lifecycle, which covers specific needs of data science. It represents the practice of collaboration and communication among data scientists and operations professionals to help manage the production ML (or deep learning) lifecycle. The movement of DataOps to MLOps and later to AIOps (19) for artificial intelligence was natural since there was technical debt.

Tom et al. (20) explain the technical debt as follows: “Technical debt is a metaphor that refers to the consequences of poor software development. Cunningham (1992), who introduced the concept of technical debt, described how ‘shipping first time code is like going into debt. A little debt speeds development so long as it is paid back promptly with a rewrite.’ Since then, the suitability of debt as a way of explaining the various drivers of increasing costs throughout the life of a software system has been affirmed by the software development community (21–25). On the other hand, debt is not necessarily ‘bad’—a small level of debt can help developers speed up the development process in the short term (21). In any case, this consequence may be felt in the longer term if the project is highly ‘geared’ (which implies onerous debt repayments), leading to slower development and killing of productivity.”

Science has been evolving to be more open, fair, and reproducible (Section 2.2.4) in the last years. Data are published on various platforms and processed in on-premise, private, or public cloud storage and services. The importance of sharing data across scientific fields has been raised.

The technical debt can thus also be considered for research. The research systems should provide functionality like data preprocessing and sharing, analytical tools, reporting tools, and a complex methodology and ecosystem that consider all those steps part of a unified lifecycle. Then, ResearchOps^1^ “provides the roles, tools and processes needed to support researchers in delivering and scaling the impact of the craft across an organization.”

**Figure 3:**
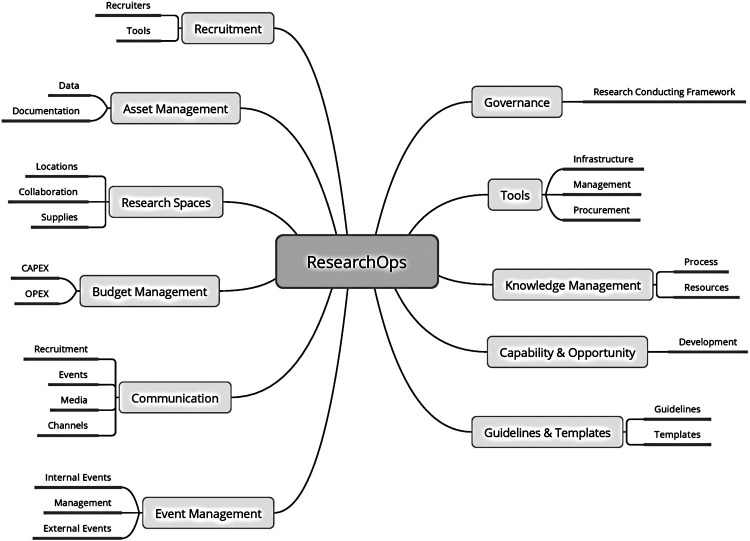
ResearchOps Community-separated resources for each of the topics. The link to community- made tools that support each aspect of these open topics can be found in the footnote below.^2^

The following topics (Figure 3) are important whenever research is managed. *Governance* defines safe, ethical, and legal research; *Guidelines & Templates* frame it formally. *Tools* are necessary for doing research, its management, and operations. *Knowledge management* deals with data and documentation (which are part of *Asset management*) and provides resources for *Capability & Opportunity* to develop career or capabilities necessary for a particular research project. *Research spaces* have to be maintained, and research staff and subjects must be recruited (*Recruitment*). This all needs to be published, promoted, presented, and advertised through the events within *Event management* and communicated (*Communication*) via various channels. All needs to be managed and maintained within *Budget management*.

Considering scientific research’s disciplines, processes, and requirements, we need to adapt existing ResearchOps or define our operational process for the health-related data lifecycle.

#### 2.1.2. FAIR principles

There is a plenitude of guidelines and principles available that can be used to store, maintain, and disclose open scientific data. However, the FAIR (an acronym for Findable, Accessible, Interoperable, and Reusable) principles (26) have become popular and widely accepted within scientific communities. They make data for computational systems easier to find, access, interoperate, and reuse, without any or just with minimal human intervention. The four intertwined categories describe how data, metadata, and resources should be described, stored, and made available to a broad audience.

The Findable principle declares that it should be easy to find data and metadata by both humans and machines. It can be achieved, for example, by assigning a globally unique and persistent identifier to data, describing data with rich metadata, and registering or indexing them in a searchable source.

The Accessible principle deals with data access since not all data have to be strictly open. If possible, metadata should be accessible even when the data are no longer available.

The Interoperable principle focuses on integrating data with other kinds of information. It is generally achieved using domain-wide agreed data formats, languages, and vocabularies. Also, qualified references to other metadata and data are included.

The Reusable principle ensures that data are easy to reuse, i.e., they can be well-replicated or combined into different settings. In this regard, data should be richly described with many accurate and relevant attributes and released with a clear and accessible license.

The FAIR principles do not state how they should be achieved; they represent recommendations that keep the data open and independent of the application domain. Multiple initiatives promote these principles across scientific fields, like the FAIR Data & Services (IFDS^3^.) or the European Open Science Cloud (EOSC^4^.).

#### 2.1.3. Terminologies and ontologies

Terminologies and ontologies are popular for modeling domain knowledge in many scientific disciplines. Roche (27) explained that an ontology is not a terminology and a terminology is not an ontology and that terminology relies on two different semiotic systems (the linguistic one, which is directly linked to the “Language for Special Purposes” and the conceptual system that describes the domain knowledge), whereas ontology does not take into account the linguistic dimension of terminology. Zemmouchi-Ghomari and Ghomari (28) state that building ontologies is considered much time-consuming and costly than building terminologies with regard to ontology complexity and formality, two major differences between these types of resources. They also claim that terminologies can be considered as preliminary attempts to model particular domains by their respective experts. Then, terminologies are intended for human users, while ontologies are mainly developed for knowledge sharing between both humans and artificial agents.

Gruber (29) formulated the definition of an ontology: “*An ontology is an explicit specification of a conceptualization*.” Borst (30) modified the definition as: “*a formal specification of shared conceptualization*.” In other words, ontologies are formalized vocabularies of terms. Le Franc et al. (31) defined ontologies in an alternative way: “*Ontologies are formal models of knowledge in a particular domain and composed of classes that represent concepts defining the field as well as the logical relations that link these concepts together*.” Designing an ontology is a long process in which it is necessary to understand the area and compile a list of used terms (and creating terminology).

The conceptualization of the real world can be also translated as taking real existing things and creating standard terms and their classes to categorize them into a hierarchical structure. However, many questions need to be answered before any description can be made.

The first question is how detailed or abstract the ontology should be since varying degrees of details are desirable. A detailed ontology can be overdefined when it describes and tracks every available detail. It can cause uncertainties when new changes are added to the ontology. On the other hand, overabstracting (generalization) leads to uncertainty in the definitions of the terms and their classes when many instances fall into more classes. The next question deals with the relations between the terms and their classes. The hierarchy and number of relationships must be carefully defined; otherwise, overdefinition or overabstracting can also occur.

Dynamic ontologies (10) add a layer for adjusting or “evolving” the ontology according to the project’s needs over time. The changes need to be accommodated once the project grows, and the used terms and relations will need to be expanded or adjusted to address these changing needs in the already existing and published ontology. Examples of such “actions” can include adding/deleting existing relations between terms, adding a new property, changing the ontologies hierarchy, and reusing certain aspects or portions of other published ontologies.

Especially domain ontologies are popular since more general ontologies are very difficult to define (suffer from overabstracting). There are hundreds of biomedical ontologies and millions of classes (uploaded to Bioportal). The list of published ontologies steadily increases.

Popular languages for the implementation of ontologies include, e.g., the Web Ontology Language (OWL) of the Semantic web or dOWL, an extension to OWL, which consists of a set of elements that can be used to model these evolutionary changes in an ontology (32).

There are a lot of web-based systems to support ontology reuse (e.g., Bioportal,^5^ OntoFox,^6^ Ontobee,^7^ Neuroscience Information Framework,^8^ and Ontology Lookup Service^9^).

Although the popularity of terminologies and ontologies is still high, the requirement for an analytical definition of the part of the world is their limiting factor. It is a time-consuming task requiring not only the definition and implementation of the terminology or even ontology itself but also its acceptance by the wider community when the ontology should become a standard formal description of a domain. This step is crucial; hundreds of existing biomedical ontologies and systems that use them illustrate this issue well. There is some hope for expanding deep learning methods, which have lower requirements for data organization and could significantly alleviate problems with overdefined ontologies.

There are many broad terminologies defined in published ontologies like the National Cancer Institute Thesaurus (NCIT)^10^ (33) describing set of terms and their relations. The main NCIT focus was on providing a controlled vocabulary used by specialists in the various subdomains of oncology. Across neuroscience, there exist projects that include terms related to event-related potentials, also containing MEG (magnetoencephalographic) or EEG (electroencephalographic) terminology.

The NEMO project (Neural ElectroMagnetic Ontologies) (34) provides an ontology that contains descriptions of classes of event-related brain potentials together with their properties, including spatial, temporal, and functional (cognitive/behavioral) attributes.^11^

Minimal Information for Neural ElectroMagnetic Ontologies (MINEMO) is the minimum set of experimental metadata required for datasets that are used in the NEMO project (35). MINEMO specifies the key information that should be provided when an ERP experiment is uploaded to the NEMO database. MINEMO terms are explicated in the NEMO ontology, a formal semantic system created for the ERP domain. There were also developed web applications (the NEMO portal) and a database aligned with the MINEMO checklist and ontology. The checklist, ontology, and database are intended to support the first complete, cross-laboratory meta-analysis for the ERP domain.

While creating new terminology (where the reuse of already existing terms is much endorsed), reusing only its essential parts may be easier than including the entire terminology. A recommended set of guidelines MIREOT (Minimum Information to Reference an External Ontology Term) (36) was created. It describes the necessary minimum of information that needs to be overtaken.

For ontologies, we have used recommendations by large and long-running standardization bodies like The World Wide Web Consortium (W3C) while including various most commonly used or recommended practices across the ontology lifecycle. It is the case with the iterative evolution, expansion, and enhancement of dynamic ontologies.

#### 2.1.4. Data formats

Storing and processing health-related data are difficult because hardware devices and software drivers usually provide data in proprietary formats. Specific neurophysiological data-storing formats can severely hamper the collaboration between the researchers, as there is a need to have the same (and usually licensed) processing tools that support these data formats (37).

The main goal of neurophysiology data standardization initiatives (38) is to create a unified data model and storage format and tools to convert existing data stored in the proprietary data formats. These standardization efforts and their results (data models/formats) can be found in the following.

In this case, we have selected a list of the data formats endorsed [Brain Imaging Data Structure (BIDS), Neuroscience Information Exchange (NIX), Neurodata Without Borders: Neurophysiology version 2.0 (NWB:N 2.0)] or submitted for endorsement (Open Metadata Markup Language, odML) to the INCF Standards and Best Practices Committee. The endorsement process consists of an expert review against an established set of criteria, a community review, and a final committee review that considers comments received during the expert and community reviews (39). As for the remaining recommended format, JavaScript Object Notation for Linked Data (JSON/LD) is one of the few data and metadata formats used by large technological giants like Google.

##### 2.1.4.1. Neuroscience Information Exchange format

The NIX data model (40) allows storing fully annotated scientific datasets, i.e., the data together with rich metadata and their relations in a consistent, comprehensive format. Although developed initially for electrophysiology data, neither the data model nor the metadata model are domain-specific. Both models can be linked to predefined or custom terminologies. It enables the user to give elements of the models a domain-specific, semantic context. In contrast to most other approaches, NIX achieves flexibility with a minimum set of data model elements. The NIX project includes native I/O libraries for C++ and Python, language bindings for Java and MATLAB, and a viewer for NIX data files, although the HDF5 (41) viewer can also be used.^12^

##### 2.1.4.2. Open Metadata Markup Language

odML is a format for storing metadata in an organized human- and machine-readable way (42, 43). It does not constrain the metadata content while providing a common schema to integrate metadata from various sources. odML facilitates and encourages standardization by providing terminologies^13^ for metadata.^14^ An example of the odML use when collecting and exchanging metadata in an automated, computer-based fashion is described in (44). Currently, the odML is included in the NIX data model.

##### 2.1.4.3. JavaScript Object Notation for Linked Data

JSON-LD (45) is a lightweight syntax to serialize Linked Data in JSON. Its design allows existing JSON to be interpreted as Linked Data with minimal changes. JSON-LD is primarily intended to be a way to use Linked Data in Web-based programming environments, build interoperable Web services, and store Linked Data in JSON-based storage engines. Since JSON-LD is 100% compatible with JSON, many JSON parsers and libraries can be reused. In addition to all the features that JSON provides, JSON-LD introduces, e.g., a universal identifier mechanism for JSON objects via the use of Internationalized Resource Identifiers (IRIs), disambiguation of keys shared among different JSON documents, a mechanism in which a value in a JSON object may refer to a resource on a different Web site, or the ability to annotate strings with their language.

##### 2.1.4.4. Brain Imaging Data Structure

The BIDS is a standard endorsed by INCF prescribing a formal way to name and organize MRI data and metadata in a file system that simplifies communication and collaboration between users. There also exists an extension onto the BIDS format called EEG-BIDS, which is specifically designed to store the electroencephalography data. If you would be interested in learning more about the EEG-BIDS format, you can find it in (46).

In both variants, it enables easier data validation and software development by using consistent paths and naming for data files. BIDS is strict regarding file organization, naming, and metadata, but to support broad adoption, it permits substantial flexibility in the details of how other dataset metadata are described within the standard (47).

##### 2.1.4.5. Neurodata Without Borders: Neurophysiology version 2.0

NWB is a data standard enabling sharing, archiving, using, and building analysis tools for neurophysiology data. NWB is designed to store various neurophysiology data, including data from intracellular and extracellular electrophysiology experiments, data from optical physiology experiments, and tracking and stimulus data (48). NWB:N 2.0 defines an ecosystem for standardizing neurophysiology data.

### 2.2. Motivation

In this paper, we focus on the health-related data lifecycle. It mainly includes data description for further processing, the richness of data/metadata from the subject perspective, correlation, and causality research. We aim to achieve that via four pillars—ontologies (2.2.1), 360-degree overview (2.2.2) of the research subject from data perspective, standardized lifecycle (2.2.3) for health-related data, and research reliability (2.2.4) through reproducibility and repeatability.

#### 2.2.1. Using ontologies

First, let us start with what are the ontologies good for (49). Their first and foremost advantage is to capture the used terminology inside any application domain (or the research subject) and map the definitions, attributes, and relations of these terms to one another. Since many terms can have multiple meanings, their precise definition helps even a newcomer to the application domain better understand the used terms and their relations; this extended vocabulary maps relations between defined terms.

An additional benefit to ontologies is that they enable easy understanding of multiple application domains and can be reused easily. Their reuse helps reduce the redefinition of the terms. When an ontology becomes widely available, it increases its value. The ontology can be expanded and corrected further down the line to a more detailed and sufficiently defined result.

#### 2.2.2. 360-degree overview

In most cases, we are talking about EEG/ERP data (50–53). These types of data bring crucial information about the measured subject, but we cannot forget to record also the data related to the subject, outside environment, and the experiment itself. These additional data provides a 360-degree overview of the measured subject and give additional potential to better understand the foundation during the analytical process.

In some literature studies (54, 55) are such data neglected, and the main focus is on neuroscientific data. It works well for narrow field research for single-purpose data collection during the experiment and making the conclusion published through single paper. However, with the greater goal, we need to collect as much data as we can, so it can be later used for multiple research use cases.

We cannot consider our work to be frontier-bringing such idea since some literature studies (56, 57) present collection of data, Metadata, and scenarios of experiments, but we would like to define standards that can be adapted as is or with extensions or adjustments.

#### 2.2.3. Standardized lifecycle

**Figure 4:**
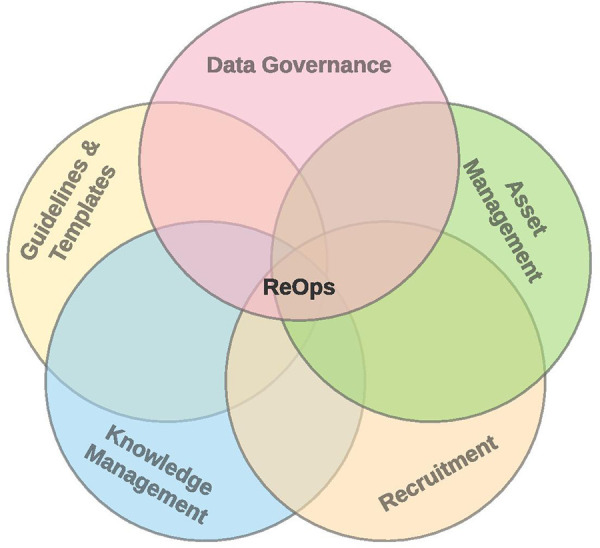
ResearchOps for health-related data.

Inspired by ResearchOps and DataOps (2.1.1), we derived the subset of disciplines useful for health-related data (Figure 4).

We kept in mind disciplines and processes necessary to cover complete neuroinformatics data lifecycle from asset management, subjects recruitment, guidelines and templates, knowledge management, and data governance.

#### 2.2.4. Research reliability

The main idea about the ontology-driven (2.2.1) system is to provide a platform for reproducibility and repeatability (58). These two major principles of scientific methods for research supporting are very important to ensure research reliability.

##### 2.2.4.1. Replication crisis

In 2005, an essay was published in PLoS Medicine by Professor John Ioannidis at the Stanford School of Medicine (59), who argued that a large number, if not the majority, of published medical research papers contain results that cannot be replicated. This is practically the foundation for later-defined term replication crisis, respective replicability, or reproducibility crisis.

The crisis itself has longer roots, but it started to be significantly used in the early 2010s (60) as part of growing awareness of the problem (61, 62).

##### 2.2.4.2. Reproducibility and repeatability

The meaning of reproducibility is to achieve the results of the experiment again with a high degree of agreement when the experiment is replicated with the same methodology by the different researchers. When the reproducibility is achieved once or several times, the experiment can be considered a valid contribution to scientific research.

The repeatability is defined as one (test-retest Reliability) of the four general classes of reliability estimates (61) from the theory of reliability (62) we know:

• Inter-rater or inter-observer reliability used to assess the degree to which different raters/observers give consistent estimates of the same phenomenon.
• Test-retest reliability used to assess the consistency of a measure from one time to another.
• Parallel-forms reliability used to assess the consistency of the results of two tests constructed in the same way from the same content domain.
• Internal consistency reliability used to assess the consistency of results across items within a test.

##### 2.2.4.3. Terms’ ambiguity

In the scientific research exists the ambiguity of reproducibility and repeatability (62, 63). The usage of the recurrent terms reproduce and replicate often means different things but sometimes interchangeable.

As (62) claims, the terminology can be classified as, First, make no distinction between the words reproduce and replicate or, second, use them distinctly. This two-term direct substitution leads to the weight issue that might be solved by various attempts to invent the terminology across disciplines and establishment of patterns that help us resolve the contradictions.

### 2.3. Summary

In the following section, we mention some of the best practices and pieces of advice that were recommended by the wider scientific audience and used within the Body In Numbers project:

• **FAIR principles**—It is the utilization of the FAIR principles across all processes, making the collected data and metadata easily accessible and shareable.
• **Size and scope of the new ontology**—It is necessary to define who will and how to use the ontology. Examples of questions that might be asked are as follows: How abstract or detailed should the ontology be? or What subjects will it cover?
• **Learning from already existing ontologies**—In case you have not much experience with creating ontologies, it is best to go through existing and published ontologies inside the same field of study when planning to create a new ontology. In this case, it is necessary to focus on how each used term is being defined and what annotation attributes are used to devise rules for the ontology creation process.
• **Appropriate annotation properties**—It is a good practice to maximize the reuse from already published ontologies. Relevant ontologies for this task can, for example, be the Information Artifact Ontology (IAO). If there are no adequate replacements, new ones can be created.
• **Naming terms**—Using plain English in the term names is strongly advised. CamelCase or Under_Score notations should be avoided. If the term has any notable synonyms or shortcuts (e.g., acronyms), they are stated. If any dedicated annotation properties are used, the rdfs:label can be used instead.
• **A unique identifier**—In the case of overtaken terms, the original unique identifier from the source ontology should be retained; otherwise, a unique ID for each new term has to be created. Organizations can generate a persistent URL that enforces the uniqueness requirement for the primary identifier, e.g., http://purl.obolibrary.org/obo/OBI˙0000185.
• **Textual definition of each term**—Textual definition needs to best describe the meaning of the term under which it is used inside the ontology.
• **Reuse (import) of external terms**—When any existing term is overtaken, the attributes and ID should be identical to the source ontology. Rules for the import of the term can be further specified inside the source ontology under the annotation property rdfs:comment.
• **Ontology open to collaboration**—Any collaboration with the community can enhance the overall quality of the ontology.
• **Ontology license**—The Creative Commons license in its latest version is advised for most of the open source ontologies. For monetized ontologies, when the ontology can be used (under which circumstances), in which projects or how to obtain access to the ontology needs to be specified.
• **Serialization of ontology**—Common formats, e.g., OWL, RDF/XML, or OBO, are defined for the publication of the finalized ontology.
• **Incremental expansion of ontology**—Certain terms might not have been properly defined as in the original plan. Small incremental additions of new terms can make the ontology overall more well-defined.
• **Data formats**—Suitable, possibly free, and widely accessible data formats are used within the tools that can be used to explore the collected data (e.g., for MRI, EEG, ERP, and more).

The methodology used in the Body in Numbers (“BiN” for short) project is mainly based on the recommendations mentioned above.

A thesaurus of all related terms used inside the project was created in the initial phase of the BiN project. The terms were related to data collection or the subsequent data processing phases. Each of these terms had the name and basic definition stated in plain English.

As a next step, these terms were separated into categories (classes of terms) and related notation properties, further used to describe all remaining terms in the ontology. An example of the annotation property is the author of the definition, textual definition, synonyms and shortcuts, and name of the term.

After this phase, it was necessary to maximize the reuse out of any already existing and published ontological sources, so that we would limit the amount of “reinventing the wheel” so to say. At this step, ontological portals were of great help (like BioPortal, OntoBee, and OBO Foundry).

In the next step, it was necessary to distinguish overtaken terms from other ontologies and newly defined terms as each needed to contain different annotation properties. In the case of overtaken definitions, all attributes were overtaken into the annotation properties equivalents of the devised ontology. For the newly defined terms, it was necessary to define a bare minimum of information that the term needed to contain (like the synonyms, known shortcuts, and textual definition). Once the process for the newly added terms was refined, a dynamic ontology was created. The ontology was then used in the next steps of the data flow.

However, the workflow for the health-related data lifecycle goes behind the created ontology. Next to the ontology, we also provide a 360-degree data overview (2.2.2). This includes our proposal for standardized lifecycle (2.2.3) inspired by DevOps, DataOps, and derived from ResearchOps. Then, we included research reliability (2.2.4) consisting of two principles—reproducibility and repeatability.

## 3. Results

In the following section, we will take a closer look onto both the abstract and also on the implementation aspect of an undertaken project from the University of West Bohemia called “Body in Numbers.” We will also describe the entire process on how the data were acquired, stored, processed, successively analyzed, and published. The unique part about this process is a specialized support module architecture developed in tandem to help with each step of the data cycle.

### 3.1. Main overview (cube)

**Figure 5:**
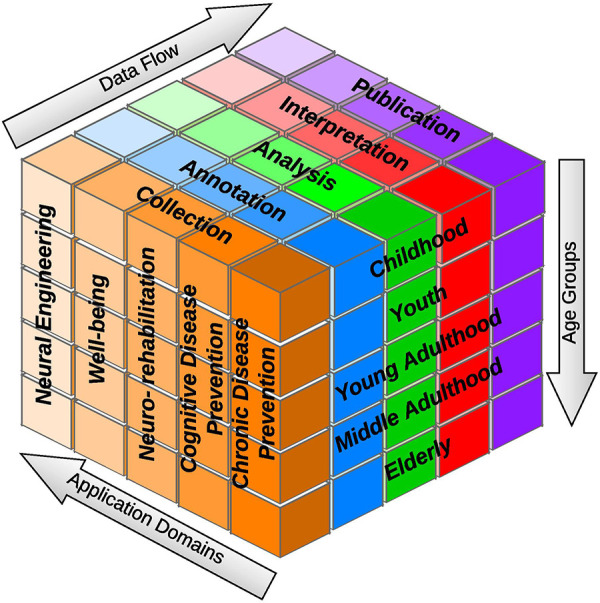
“Cube” overview: each cube part can be imagined as a box with specific needs, which have to be accounted for and supported by the underlying system.

The implementation of neuroinformatics experiments is a conceptually complex system that solves every aspect of the data flow in races to a given category of interest within each of its functionalities. In this case, it is the age category of the participants in the experiments. The analytical model of the system is shown in Figure 5. This system determines any way, for example, how the collected data from children and adults are handled. Other variants of research addendum agreements and questionnaires could be used to measure children and adults. These templates are used to change measurement procedures depending on whether a child or an adult participates in the experiment. Later in the same year, a new change needed to be implemented related to the analysis, namely, our goal was to find a statistical significance between the already collected data and metadata.

When it comes to evaluation of the efficacy in the presented lifecycle (in the form of the “Cube”), the chosen dimensions separate the larger process into small tasks that can be at least partially (based on the situation) automated outright, or it is possible to create support processes that overall make the step easier (or faster) across the data flow. For example, data quality can be ensured during the data collection process by filling out all mandatory fields and pairing included metadata. Data duplication can be prevented (at least partially) in this step. As for the mentioned support processes, the analysis part of the data flow can automatically prepare overview statistics about the collected data that help classify or further analyze the data and metadata.

The chance of errors in the filled-out data and metadata is dramatically decreased when steps eliminate the “human factor” from highly repetitive or easy-to-automate tasks. Aside from that, the time necessary to spend in each data flow step will also decrease, with much of the validation steps being eliminated from the equation.

Some of the proposed metrics for evaluating this lifecycle were defined as follows:

• percentage of complete data entries inside the data collection phase for the dataset,
• percentage of described data entries inside the annotation phase for the dataset,
• number of newly required metadata definitions (number of new terms) from the dataset, for inclusion into the ontology,
• time spent in each of the data flow phases, and
• age range histogram for all measured participants inside the dataset.

#### 3.1.1. Data flow

This dimension helps streamline and potentially automate repeatable experiments, minimizing room for errors and increasing overall efficiency. This part improves the description of the experiment. Experimenters can make quicker and smarter decisions. Researchers are empowered to collaborate in a more productive and agile way.

The data flow phases are shown in Figure 5. The collection phase deals primarily with acquiring data from a BiN project participant; the data are subsequently preprocessed (more details are in Section 3.3).

The preprocessing consists of cleaning the data and adjusting it for the next step by converting it into the format expected in the analysis phase.

The analysis phase is currently aimed at determining statistical significance between each segment of the measured data, the values achieved by participants, and respective questionnaire answers. The summary tables and graphs are created and used for finding further subsequent research activities.

During the interpretation phase, new hypotheses are outlined and revised by scientists to refuse or confirm them.

In the last phase of the data cycle, anonymized data are published utilizing raw data. The results obtained from the initial analysis and the pilot experiment are included. The data published in this way help the broader scientific community answer further questions and hypotheses.

#### 3.1.2. Application domains

There already exist conceptually close domains. The Chronic Disease Prevention (4.2.2) domain is focused on nutrition counseling. The food balance system is calculated to reduce the user’s food consumption. This system can monitor the users using smart bracelets (such as FitBit) when information is sent to a mobile tracking application.

The Cognitive Disease Prevention (4.2.2) domain works with cognitive games that improve memory, attention, speed, and problem-solving abilities.

Both domains are indirectly related to brain activity data (e.g., EEG, ERP), which however can be used to further help with either of the previously mentioned application domains.

#### 3.1.3. Dimensions (age groups)

The project focuses on the age range from 11 to 60 years. Beyond this age limit, the measured results are distorted by specific errors related to either young or advanced age. In the case of preschool children, there may be problems with the numbers (color vision, number recognition) and hand reaction times (the children might be unable to reach the upper buttons on the table due to the minimum required height). In older age, the problems may be responding quickly to stimuli using legs (leg reaction times) or hands.

The monitored age categories are shown in Figure 5 (age groups), namely, childhood, youth, young adulthood, middle adulthood, and the elderly. Of course, the consent and completion of the questionnaire for a young child will be different and proceed differently than for adults (in the case of young children, their legal representatives or parents must approve the participation in the project and The General Data Protection Regulation data processing).

### 3.2. Data flow dimension

#### 3.2.1. Data flow semantics

The first step in data preprocessing is to identify the individual parts of interest and categorize them so that the data are transferable and shared between different working groups. Creating the ontologies schematizing relations between each part of the cube is necessary to make the data more shareable. Therefore, the task was to develop a system that would be able to preprocess the data and make it easier to share with members of the scientific community. The project aims to create a uniquely annotated collection of heterogeneous health-related data available for further analysis. The Body in Numbers system helps collect additional metadata from questionnaires combined with the measured health-related data (e.g., weight, height, and blood pressure) and EEG data from brain–computer interface (BCI) experiments.

The data collected are anonymized and published within data articles. They are converted into one of the commonly used RDF formats, and a backing ontology is created; it may define additional properties.

The Body in Numbers terminology has included and used the best practices presented in Section 2.3. The main set of terms was defined (the definition under which it is used in the BiN terminology) and compared against the existing definitions from relevant sources. If the term was already described inside other ontologies, but the description did not match the meaning utilized in the BiN project, a new definition of the term was created. Otherwise, the existing definition was overtaken, and a citation was attached appropriately.

**Figure 6:**
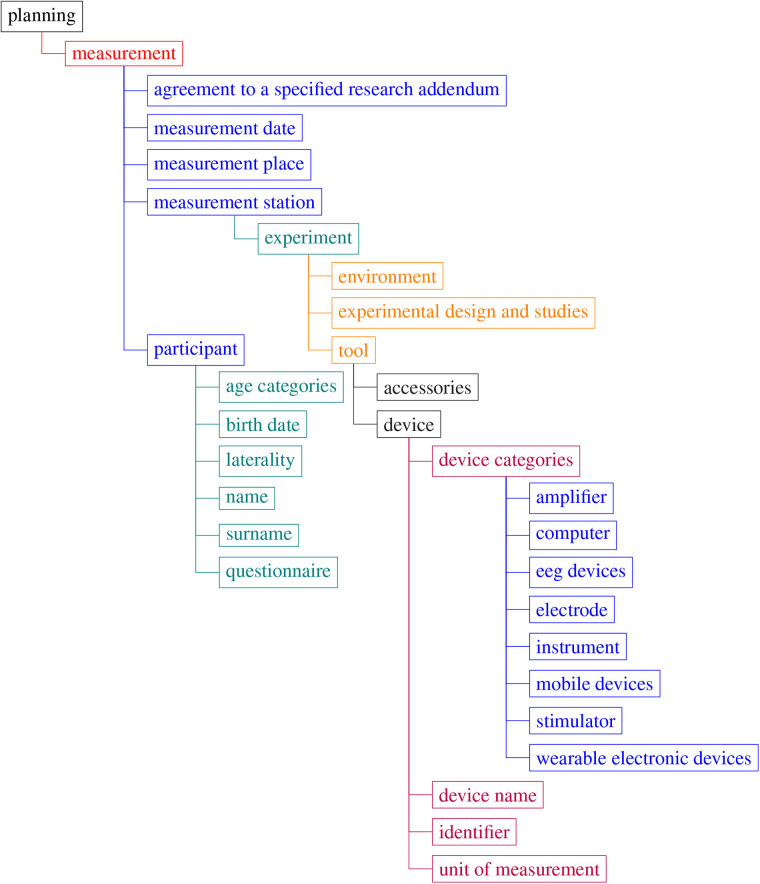
Body in Numbers terminology—examples of the root terms. Note that tree visualization only contains a few top degrees of the tree. For more details, see the link to the terminology in Section 3.2.2.

The basic set of key terms are given in Figure 6.

#### 3.2.2. Data flow detail

**Figure 7:**
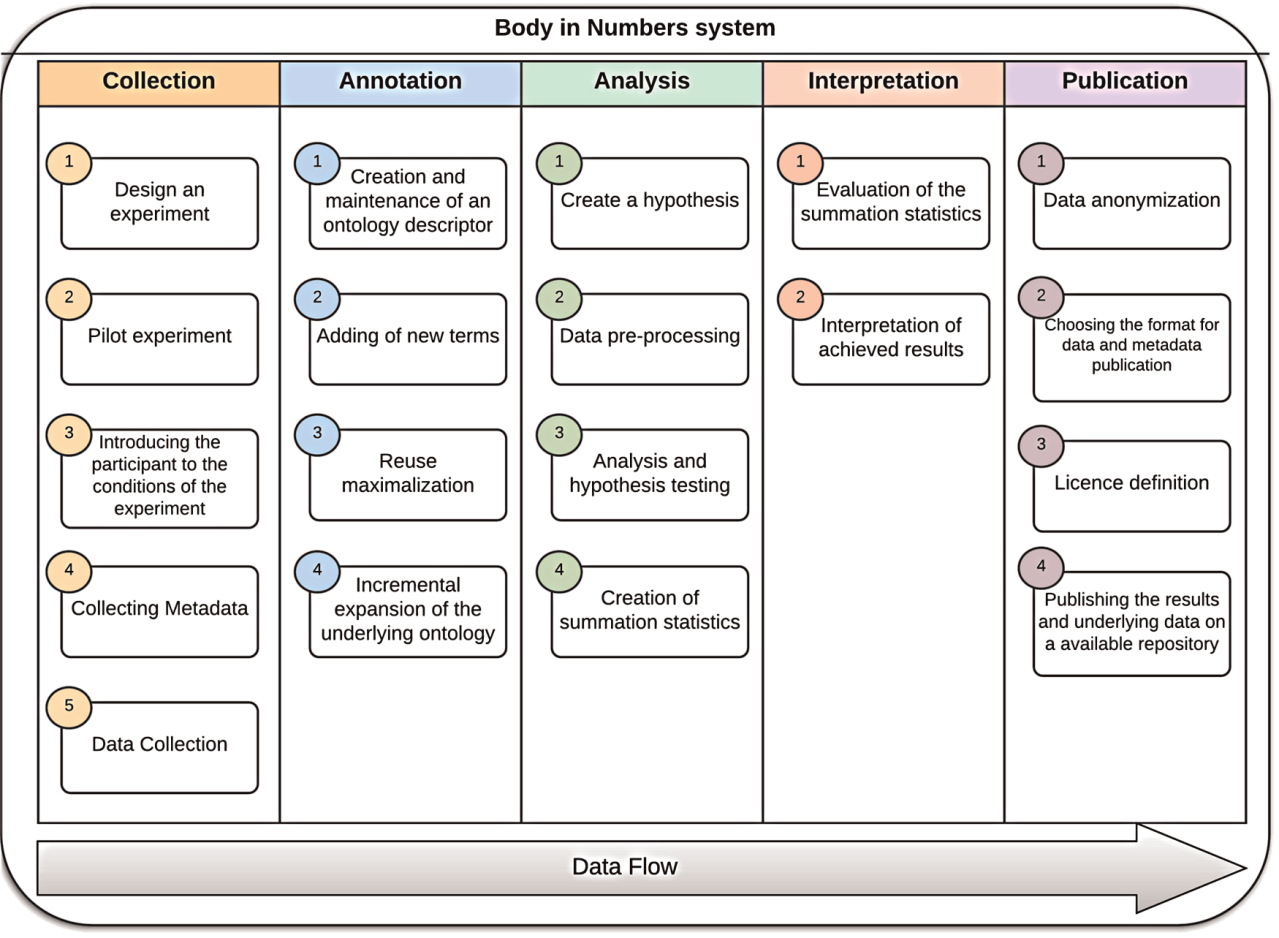
Data flow–layer details.

Considering the cube as a modular representation, we sliced its particular layers with the following topics (Figure 7).

Body in Numbers (BiN) uses its specific terminology:

1. Tools—Devices and tools used during experiments (e.g., pressure gauge for measuring pressure and pulse, and spirometer for measuring lung capacity)
2. Experiments—Participants examination (e.g. measuring blood sugar requires a finger prick by taking blood on the measuring strip and then evaluating the results with a glucometer; the experiment’s output is blood sugar = 6.4 mmol/l).
3. Locations—A locally determined measurement that may contain one or two more experiments.
4. Measurements—Definition of all sites, experiments, and assigned aids.
5. Scheduling—Definition of what (e.g., glucose level, weight, height), how (e.g., glucosemeter, scale, meter), and what (specific type of the measuring instrument, i.e., specific glucosemeter type) is used for measurements.

**Figure 8:**
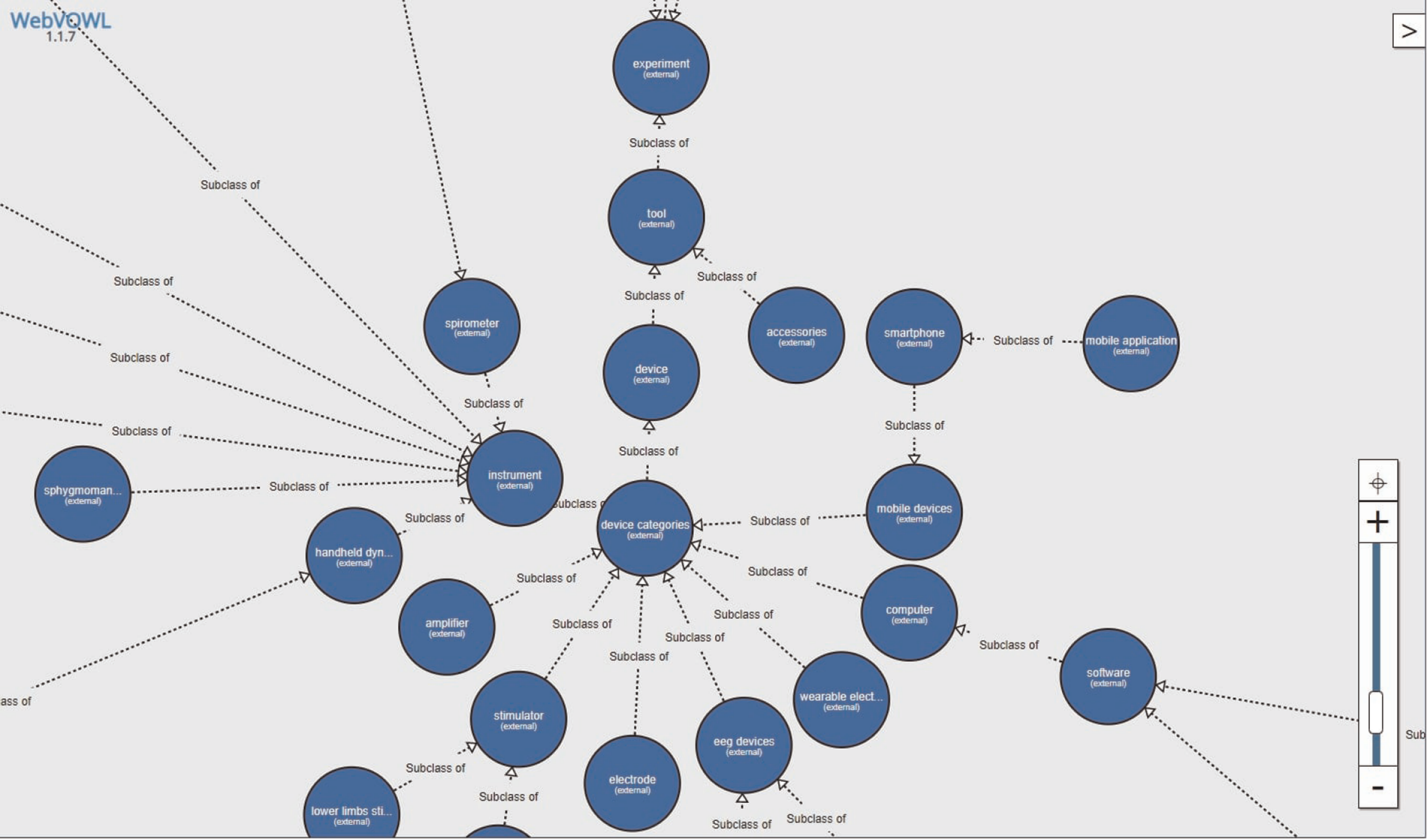
Body in Numbers ontology—the parent node “Device categories” contains links to its child and sibling nodes. Only the subclasses are visualized (without any additional defined properties). The ontology was visualized with WebVOWL: Web-based Visualization of Ontologies, version 1.1.7, available at http://vowl.visualdataweb.org/webvowl.html.

The created ontology^15^ contained 141 classes of terms, of which 56 classes of terms were overtaken from already existing ontologies (with their original definition), and for the remaining 85 terms, new definitions were created (the definitions from existing published ontologies were insufficient or the terms were not yet defined). The ontology also contains 30 annotation properties where every single one was overtaken from existing sources. An example of subclasses visualizations is available in Figure 8.

##### 3.2.2.1. Data collection

The following subflow is typical for the data collection process:

1. The experiment and its environment (Section 3.2.2.2) are defined.
2. The experiment is introduced (64) to each participant; they sign the consent agreement.
3. The participant is registered to the system [(64), more information is available in Section 2.2].
4. The participant fills in the questionnaire [(64), more information is available in Section 2.3] related to a particular measurement or multiple measurements.
5. The measurement is performed.
6. The collected data are stored (Section 2.1.4) in a standardized format together with their metadata collected during the registration and questionnaire phases (Sections 3.3.2 and 3.3.4).
7. Data analysis is performed (Section 3.3.3).
8. The raw data are prepared for publishing (Section 3.3.5).

At the beginning of the experiment, the participant is acquainted with the project’s goals and the necessary conditions under which the data are used, and if they agree, the questionnaire part of the investigation is filled in. After completing these steps, the participant completes the selected (or all available) measurement sites. The collected data are then exported into a .csv or .xlsx file, further used during the preprocessing and later in the statistical module. After the unification and purification of preprocessed data, an analysis follows. In this study, graphs of interest [age, body mass index (BMI), and others] and chosen statistical parameters are used to evaluate statistical dependencies and whether they are essential. From the statistics compiled, signs of some interesting dependencies can be found.

##### 3.2.2.2. Environment

The measurements can occur in various environments with limited control of outside disturbances. It affects possible (and even substantial) modifications of the experiment. The typical environments are as follows:

• **Hospital environment**—The hospital operation’s limitations and the participant’s health status are usually significant; the experimental procedure needs to be adjusted to these limitations.
• **Laboratory environment**—The laboratory environment is generally highly controlled; only minor modifications of the experimental procedure are usually required.
• **Participant home environment**—The most suitable environment for the participant; usually minor to averaged modifications of experimental procedure are required.
• **Public environment**—A high range and variety of unwanted disruptions are present; these create unwanted side effects—substantial and *ad hoc* modifications of the experimental procedure are common.

The environment is defined within the Body in Numbers system as a part of the measurement site.

##### 3.2.2.3. Annotation

While recommendations and opinions on what a proper ontology should contain are widespread, there is no unified opinion on what each defined ontology term should contain (in terms of granularity and detail).

The authors of ontologies generally do not use identical defining terms, and ontologies often contain similar definitions of synonyms even inside the same field of expertise. Functionally identical terms, like a term defining the author of a dataset, can be marked differently in each ontology (e.g., author, original_author, creator, and more).

Consequently, global organizations (such as OBO Foundry, Open Semantic Framework, or W3C) bring recommendations and standardization efforts. These standardization efforts include, e.g., rules that help define importing procedures for terms already defined in published ontologies, the necessity to define nomenclature, and providing text definitions for each contained term inside the ontology.

##### 3.2.2.4. Analysis

In analysis, we created a combination of hypothesis testing and basic data overview variables. The basic data overview consisted of summation values like the number of artifacts within the EEG dataset, averages of various kinds, e.g., box plots of BMI separated by specific categories, reaction times of either upper or lower limbs, and age intervals. Another part of this information also consisted of more EEG data-related variables, mainly the P300 visibility, detection, and latency.

##### 3.2.2.5. Interpretation

A statistically significant relationship was examined between the metadata (questionnaire) and the measured data. For example, if a participant answered that he/she enjoyed physical activity, he/she was supposed to answer faster when performing the reaction time experiment.

When performing statistics, we might find repeated patterns in the measured data. If such patterns are identified, it is worth studying what is causing them to appear. A typical example of a searched pattern is the P300 component, which is prominent during visual stimulation in most circumstances.

##### 3.2.2.6. Publication

During data publication, an open and widely accepted data format for data storage and the availability of tools to work with the collected data are considered. For example, table-like data are suitable to be published in CSV or Excel Spreadsheets (rather than in RDF). EEG data are preferred to be published in an open format, like NIX, rather than in a proprietary format.

### 3.3. Body in Numbers

**Figure 9:**
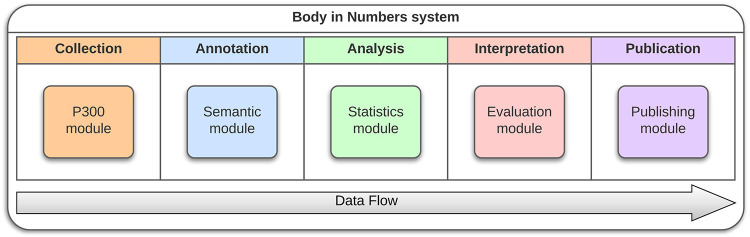
BiN lifecycle—For each of the data flow steps, module was developed that assisted with their respective tasks independently of the remaining architecture.

We will now show a practical example how the project Body in Numbers system was developed to satisfy the previously defined abstract necessities (i.e., the “Cube”). Initially, we needed to design a system for fast health-related data collection. This can be partially remedied by an individualized exercise and wellness program that integrates basic knowledge domains: lifestyle, sports and fitness, nutrition, and personal/environmental health. However, collecting, managing, and analyzing data and metadata related to these domains is demanding and time-consuming. Moreover, the appropriate **annotation** of raw data is crucial for their subsequent processing. A proposed software infrastructure included the P300 module, a specialized module for data collection of ERP data (3.3.1), a subsection for **data collection**, a semantic module (3.3.2) for **data annotation**, a statistical module (3.3.3) for **data analysis**, an evaluation module (3.3.4) for **data interpretation**, and a publishing module (3.3.5) for **data publication**. A part of this infrastructure, namely, the P300 module, was developed and tested outside laboratory conditions. This software prototype allows experimenters to collect various heterogeneous health-related data in a highly organized and efficient way. Data are then evaluated, and users can view relevant information related to their health and fitness (65). The structure of these specialized modules is available in Figure 9.

#### 3.3.1. P300 module

**Figure 10:**
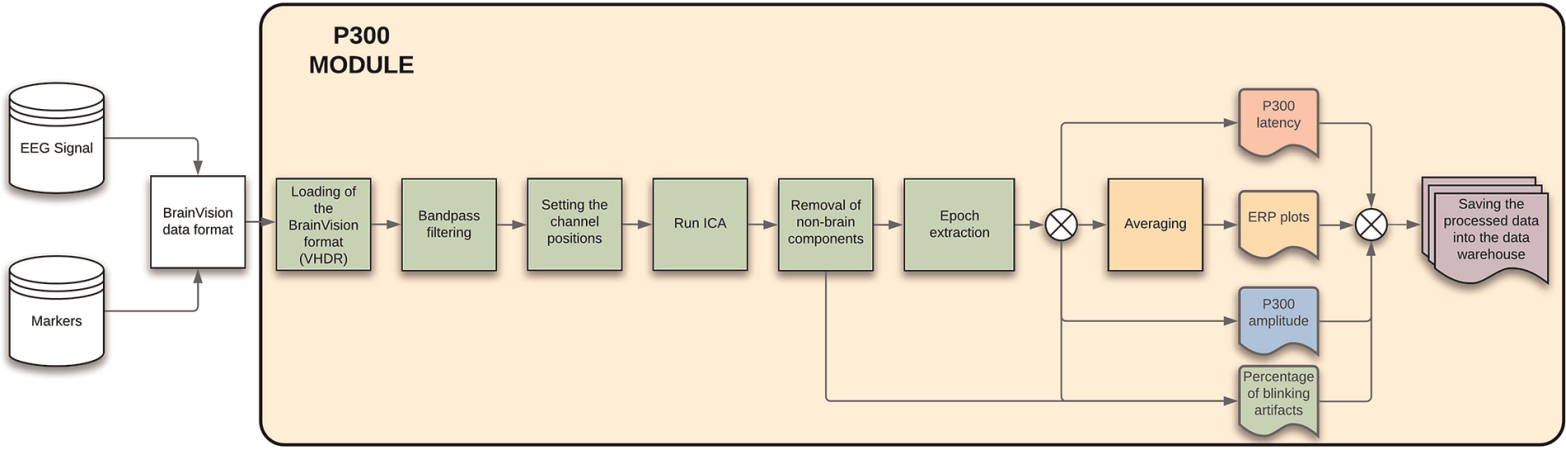
P300 module—The figure describes each of the steps that will be done automatically by the module, showing also the process inputs and outputs.

The P300 module provides basic information and statistics (like noise percentages from whole dataset and average values, i.e., response time) related to the EEG signal. When the EEG signal is cleaned and preprocessed, the P300 components latency and amplitude are extracted, with plots of averaged ERP components and blinking artifacts. The details can be seen in Figure 10.

#### 3.3.2. Semantic module

The semantic module ensures that the BiN ontology is updated when needed. This is achieved by varying approaches based on whether the newly encountered term is already known.

The data are enriched based on the used ontology if the term is known. For example, a list of measurement sites with the used devices is created; when heart rate is measured, both the units and device type are automatically added.

**Figure 11:**
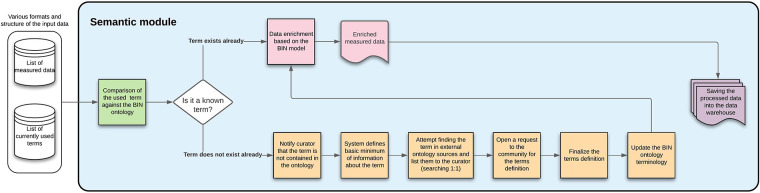
Semantic module—the figure describes each of the steps that will be done automatically by the module, showing also the process inputs and outputs.

If the term is not known, a curator has to decide whether a definition for this new term already exists or if a community poll approach needs to be initiated (for the most useful and up-to-date definition). Both of these approaches eventually define a new term for the BiN ontology. This module description is available in Figure 11.

#### 3.3.3. Statistical module

**Figure 12:**
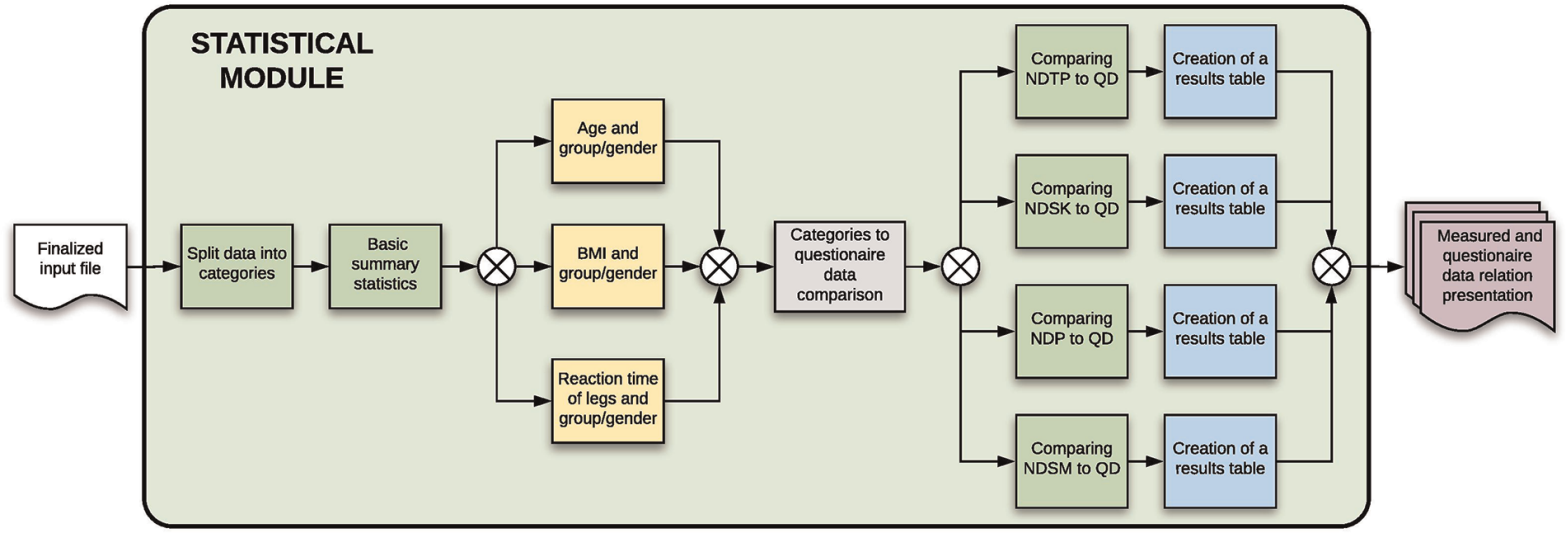
Statistical module—the figure describes each of the steps that will be done automatically by the module, showing also the process inputs and outputs.

The statistical module looks for statistical significance between the metadata (questionnaire) and the measured data. The data are first preprocessed and split into sections (based on the used questionnaire), which are then compared against each category separately using a stepwise regression on a 5% significance level. The statistical module is further described in Figure 12.

For example, if there is a question about sport in the questionnaire, the stepwise regression will show it as statistically significant to participant’s reaction times (both hands and legs), as the reaction times are shorter when the participant is doing some form of a sport.

#### 3.3.4. Evaluation module

**Figure 13:**
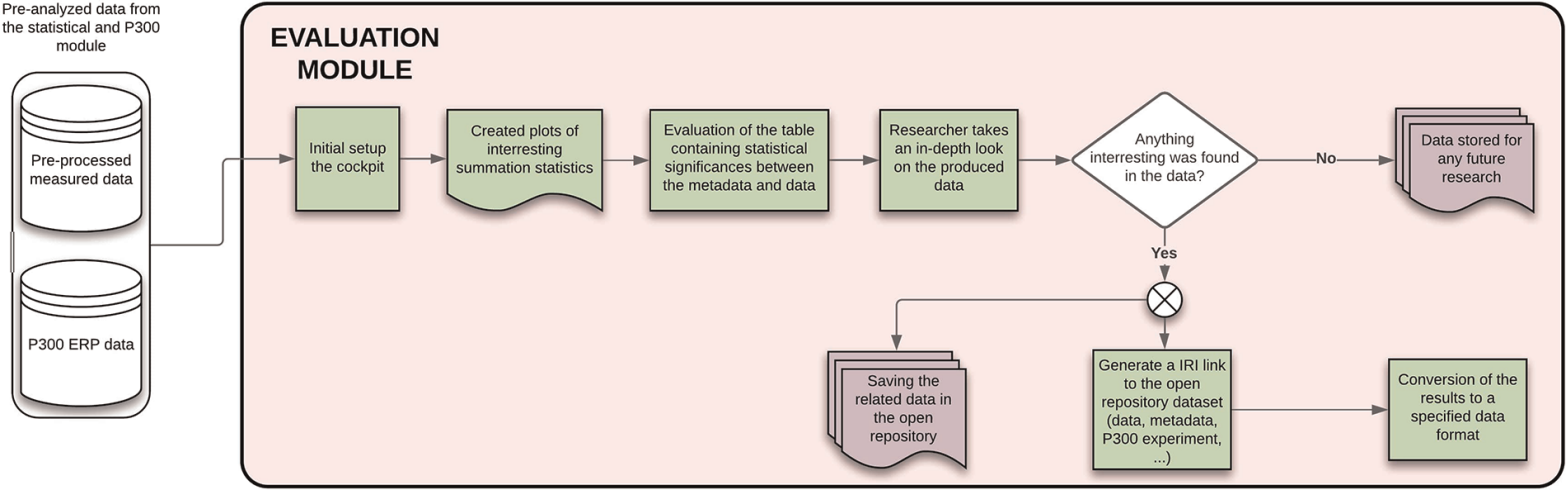
Evaluation module – The figure describes each of the steps that will be done automatically by the module, showing also the process inputs and outputs.

The resulting graphs generally contain averaged or summed statistic values, for example, top five fastest participants’ reaction times, average reaction time, number of participating smokers vs. nonsmokers, etc. If the researcher finds anything interesting they would like to publish or further investigate, they can let the system create an ID link to the dataset and, if necessary, convert the measured data/metadata into a preferred format. Details of this module are available in Figure 13.

#### 3.3.5. Publishing module

**Figure 14:**
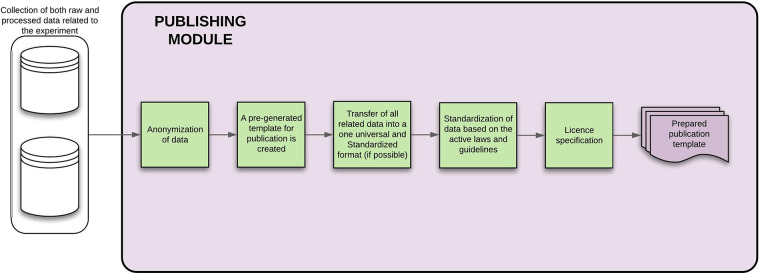
Publishing module—the figure describes each of the steps that will be done automatically by the module, showing also the process inputs and outputs.

The publishing module is mainly responsible for the anonymization of data that are going to be published within a journal and creates a LaTex template that is filled directly with the measured data, attached to the template, or linked with the generated ID from the evaluation module. A license is stated (open or closed variant) within this template. The output is then for the researcher to be further filled out. This module is shown in Figure 14.

### 3.4. Dimensions

The 360-degree overview (Section 2.2.2) is beneficial for further data processing and analysis. It gives context to health-related data sources and allows researchers to work with them from different perspectives, e.g., filter them to get a significant subset or cluster them into different cohorts.

What kind of sets of data and metadata would be useful to collect as part of the undertaken experiments have been discussed extensively. In our case, we have looked for useful sets of metadata recommendations to collect in similarly undertaken experiments.

This EEG experiment (66) was aimed mainly at motor imagery BCI; additional metadata were collected to be used for further analysis and dataset evaluation. Recommendations about what metadata could be helpful as a part of an EEG dataset were described by (67) back in 2000.

The above-mentioned literature considers the following dimensions to be the most significant:

• age groups,
• gender,
• handedness,
• BMI,
• eye defects, and
• geolocation.

For example, brain neuroplasticity changes with age. Older people are more likely to have problems with some of their motor skills (and the reaction times of either hands/legs grow as a result). Most of the collected data are more precisely evaluated based on age groups instead of age.

Gender was chosen instead of the “easier-to-manage” sex. Handedness (which hand is used by the participant in most tasks) is more valuable than laterality (superior development of one side of the body or brain).

BMI is valuable as it can be used partly to distinguish between the more bodily challenged participants, as these generally have a higher chance of being affected by chronic diseases. Eye defects are essential since most of the experiments relied on visual stimuli.

Participants feel more comfortable under different weather and temperature conditions (hot, cold, humid, or dry environment). Even the measurement’s geolocation affects the data quality and the participant’s physical and mental condition. The uncomfortable outside environment leads to more artifacts in the measured data. The participant’s home can positively affect his/her mood and concentration.

## 4. Discussion

This part discusses how the Body in Numbers system can be used as a template to create an instance of the real system. Further modules of the system are also described.

### 4.1. Validation use case

The Body in Numbers system was initially designed to rapidly collect heterogeneous health-related data for chronic disease prevention (obesity, diabetes). The original idea was first published in 2017. However, as the project has grown, the system expanded from collecting pure data to accommodating cognitive and chronic disease prevention and started to relate to neurorehabilitation and BCIs.

As the number of subjects has grown, the need to have well-annotated data has arisen. It would allow for creating different datasets based on the investigative needs (e.g., creating a study covering the youth—a need to narrow down the dataset to only people between 15 and 24 years). The earliest Body in Numbers system has been expanded to accommodate other data dimensions and has allowed researchers to collect and analyze data in different environments.

A working example of such a system, accommodating a wide range of functions, is shown.

#### 4.1.1. Proof of concept

As the next step, we defined, collected, and annotated human reaction times and relevant health-related data and metadata for further human physical and cognitive performance analysis. A collection of human reaction times and supporting health-related data was obtained from two groups comprising together 349 people of all ages—the visitors of the Days of Science and Technology 2016 held on the Pilsen central square and members of the Mensa Czech Republic visiting the neuroinformatics lab at the University of West Bohemia. Each provided dataset contains a complete or partial set of data from the following measurements: hand and leg reaction times, color vision, spirometry, electrocardiography, blood pressure, blood glucose, body proportions, and flexibility. It also provides a sufficient set of metadata (age, gender, and summary of the participant’s current lifestyle and health) to allow researchers to analyze further.

A well-annotated collection of human reaction times and health-related data suitable for further lifestyle analysis and human cognitive and physical performance was provided. This data collection was complemented with a preliminarily statistical evaluation. A procedure for efficiently acquiring human reaction times and supporting health-related data in nonlaboratory and laboratory conditions was also presented (64).

Thanks to the success and daily use of the system, new requirements related to better security, Scalability, and maintainability of its architecture have emerged. The next work presented advances and changes in the architecture of the Body In Numbers health strategy framework, mainly focusing on a new definition of user roles, optimization of the system deployment, and orchestration of the system components. A Kubernetes cluster prototype was used as proof of concept to demonstrate the improved architectural solution (68).

### 4.2. Application domains

Relations between the person’s daily life and predispositions toward specific health-related issues have been proven (e.g., (69) that covers health-related topics related to insomnia, (70) for the noise sensitivity and its effect on health, and (71) for the movement amount and its impact on health). Most well known are the issues connected with jobs with minimal movement or jobs that heavily impact a person’s body.

The question of prevention is, most of the time, a decisive factor in evading any health-threatening problems that might occur, based on the person’s daily lifestyle when aging. The topic of prevention is vast; five notable topics were selected:

• **Chronic disease prevention** (4.2.1)—it is related to recommended movement activities throughout the day (generally concerns jobs with large amount of sitting).
• **Cognitive disease prevention** (4.2.2)—it is related to maintaining healthy mind and cognitive abilities
• **Neurorehabilitation** (4.2.3)—when an accident happens that hinders brain functions (and subsequently, e.g., speech and mobility), neurorehabilitation helps with the person’s recovery.
• **Overall wellbeing** (4.2.4)—chatbots help reduce the overall effect of depression symptoms.
• **Neural engineering** (4.2.5)—it is a discipline aimed at creating Brain–computer interfaces to control machines by using a person’s mind.

#### 4.2.1. Chronic diseases prevention

Smoking, excessive drinking, overeating, and physical inactivity are well-established risk factors that decrease human physical performance and increase the incidence of chronic diseases. Moreover, epidemiological work has identified modifiable lifestyle factors, such as poor diet and physical and cognitive inactivity, associated with the risk of reduced cognitive performance (72). Chronic diseases present an enormous burden to society by increasing medical costs and human suffering. The Body in Numbers system aims to influence such modifiable lifestyle risk factors in voluntarily enrolled individuals, thus decreasing the incidence of chronic diseases.

The Body in Numbers system enables the collection of large amounts of heterogeneous data and related metadata in relatively short periods, enabling repeated measurements of participants, data processing, and evaluation. The system output is a list of information for participants (recommended exercises) and management (employees in various physical “fitness” categories).

This service is provided to firms and institutions in a series of steps:

• Participants are registered into the system.
• The participant fills in the entry questionnaires containing questions aimed at food consumption habits (dietary and drinking habits), current lifestyle, significant health issues (diseases), intensity of medical checkups, smoking habit, and the intensity of smoking, whether or not the person is currently under stress and subjectively quantifies it, quantity and quality of sleep and more (filling out the questionnaires takes about 3–5 min).
• The system organization allows to manage 16 participants at once (they are eight measurement sites).
• The output from this system includes raw data (e.g., participants’ blood pressure, cholesterol levels, and BMI), and the participants’ categories based on their willingness to participate in exercises.
• The participants receive advice on which exercises are most beneficial to them individually and a set of eating habit adjustments (based on their favorite food instead of replacing them with something different).
• Currently, all these properties and calculations were evaluated based on the Czech Republic Healthcare System using the standardized SI units and measurements.

#### 4.2.2. Cognitive disease prevention

The Body in Numbers system also helps with cognitive impairments by offering “brain games,” which motivate users through various memory, attention, visuospatial perception, language and speech, or problem-solving exercises. These games are primarily intended for mobile devices, as the exercises can be done almost anywhere with little effort and setup time.

Brain games can be adjusted to the user’s experience and needs. Some of these games can also be used with a special neurogear (the NeuroSky Mobile headset).

In the “Dangerous path” game (one of the memory-oriented exercises), the user has to memorize the location of “good” and “bad” objects at the beginning of the game. After this initial startup phase, the path is obfuscated by darkness, and the user needs to find a way from start to finish while evading all the “bad” objects.

The “Save the princess” game uses additional gear. The user is equipped with the NeuroSky Mobile sensor, which measures the levels of concentration and meditation. The goal of this game is to save a captured princess somewhere on the mobile screen while destroying “bad” objects through the usage of a cannon that shoots in a straight line within a time limit. The catch is that the shot from the cannon is only as strong as the users’ concentration level. With low concentration, shooting the “bad” objects might be necessary multiple times.

The primary goal of these games is to motivate users to challenge their cognitive skills throughout short repeated sessions, working as a preventive measure.

#### 4.2.3. Neurorehabilitation

In neurorehabilitation, it is necessary to adjust the exercise to the needs of each user to motivate them for repeatable sessions. This can be generally achieved by appealing to the user’s hobbies and dynamically adjusting the rehabilitation task’s difficulty level.

The “Smart Train” model was created in the Body in Numbers project. This is a handcrafted railway model that manipulates the train using the BCI. The user drives the train using a NeuroSky Mindwave Mobile headset (similar to the previous “Save the princess” game). The supported operations are as follows:

• The speed of the train model is affected by the concentration level achieved in the last 3 s (numeric average); this selects one of the four defined speeds.
• Change of direction (achieved by blinking twice in succession within 2 s).
• The train is stopped by achieving a meditation level of 100%.
• Lights on the train are turned on/off by blinking once.
• If 80% of concentration is achieved, the train starts to hoot.
• If the locomotive is kept stopped for 10 straight seconds, the conductor starts whistling.
• When the headset signal is classified as good, the locomotive starts up and starts to move based on the concentration levels.
• If the headset signal is low (e.g., due to poor electrode contact with skin), then the locomotive stops and turns off the engine.

The “Smart Train” neuroexercise challenges include stopping the train at a specific location (e.g., on the boarding platform) or achieving the maximum speed for a certain number of seconds. The rewarding motivational aspects of this exercise (“locomotive hooting,” “conductors whistle,” and “locomotive lights”) reward and inform the user about the current state of the concentration levels and blinking. Also, the effort required to achieve high concentration levels can be modified manually inside the application, making these tasks easier or demanding based on the user’s current needs.

Another example of a rehabilitation exercise is a ball being moved using a controller and a NeuroSky Mindwave Mobile headset. The direction of movement is set using the controller, and the speed is controlled by the users’ concentration levels measured by the headset. In this case, the goal is to navigate the ball through a modifiable labyrinth of passages as fast as possible.

#### 4.2.4. Overall wellbeing

In recent years, wellbeing has been an interesting topic for scientific research. Wellbeing fits various applications, from healthy eating to mindful living. Such services can be easily provided by various applications that notify the user of repetitive activities or maintain healthy habits. When the repetitive notifications become annoying, there is a chance to increase adherence and attrition by using gamification or psychology in natural language conversation.

The natural conversation can be scaled and automatized through dialog systems called chatbots. For example, a food tracking chatbot named Nombot (73) was developed. The dialog systems Woebot (74), Wysa (75), and Youper (76) serve as a treatment for people with symptoms of depression and anxiety. Lark (77) was designed to promote weight loss and other health behaviors related to diabetes prevention.

The approaches differ; Woebot utilizes gamification with various motivation types like points collection or higher-level unlocking. The remaining two are built on top of cognitive behavioral therapy. It combines behavioral techniques with cognitive psychology, the scientific study of mental processes, such as perception, memory, reasoning, decision making, and problem solving, to replace maladaptive behavior and faulty cognition with thoughts and self-statements that promote adaptive behavior (78).

Furthermore, although these dialog systems are far from the perfection of full human intervention, they are simple to use and available 24x7 with significant results, for instance, in the reduction of depression symptoms with randomized controlled trials (74).

In the Body in Numbers project, a wellbeing module is under development. It is interconnected with various projects mentioned above like “Smart Train” and investigated within research activities (79).

#### 4.2.5. Neural engineering

The created BCI ERP Experiment, the “Guess the Number,” uses a visual stimulation where the participant picks one number between 1 and 9 and focuses on it throughout the experiment without telling the experimenters (e.g., this number effectively becomes the target stimulus). In this case, the experimenters must correctly guess the number the participant thought.

Throughout the experiment, the participant is exposed to single pictures of each number between 1 and 9 (shown in random order) on the screen while the EEG signal and stimuli markers are recorded. Concurrently, experimenters observe average ERP waveforms for each number, search for the P300 component, and try to guess the number thought. During this time, the same “guessing” is done with a software component, which automatically identifies the number thought. The guess is verified at the end of the experiment when the participant is asked to reveal the number thought (80, 81).

A variation of this experiment was also done with a picture of nine tasks (e.g., opening a window, eating, calling the nurse), where instead of the numbers, one of the tasks was being used as the target stimulus. This variation was initially created to demonstrate the use of BCI to the public while also serving users with limited mobility (e.g., people who cannot move their bodies freely and cannot talk) to communicate or accomplish some basic tasks.

### 4.3. Experience with the implementation of best practices

The devised Body in Numbers ontology was successfully overtaken and used within the components of the Body in Numbers project while maximizing the reusability of existing ontologies. The same was applied to the overtaken terms and the annotation properties, where all the properties were reused from existing ontologies. The resulting ontology was published on the Bioportal for community reuse and in various data formats: OWL, CSV, and RDF/XML.^16^

So far, the current use of the ontology is limited to the team of Body in Numbers, which accounts for only tens of people at a time. The community feedback is severely limited, even though the incremental processes for expanding the existing ontology are in place.

### 4.4. Future work

The following works are planned:

• Extension of terminology and ontology used in other application domains (cognitive disease prevention, neurorehabilitation, overall wellbeing, and neural engineering).
• Completion of individual application domains (like neurorehabilitation, overall wellbeing and neural engineering), data collection, and involvement of machine learning in the evaluation of results.
• Expansion of the used terms based on community feedback.
• Increase in the number of indexers on which the ontology is available.

It is clear to us that this paper touches on the current absence of best practices in the health-related data lifecycle and presents, in particular, technical and methodological solutions that contribute to the sharing of annotated data from different providers. However, we have not practically touched on the nontechnical issues that accompany the possible use of these best practices.

In general, using best practices that contribute to reproducible data collection, annotation, analysis, interpretation, and publication is a laborious process that requires extensive knowledge and community involvement from all stakeholders. The resulting rewards (i.e., any additional benefits from providing well-described and reproducible data and results) are often perceived as remote and uncertain. Such a process is then necessarily more compromised in its implementation. Thus, the acquisition of clean or well-annotated data (there is no substitute for clean data), the use of shared terminologies, ontologies, and data formats (sharing them contributes to mutual understanding; AI methods better handle extensive data collections), or software and data engineering practices (they contribute to reducing technical debt) are goals that can only be gradually achieved if the environment supports and rewards such efforts. Providing best practice methods, resources, and tools for the health-related data lifecycle presented in this paper is one support in this effort. Another important support may be a gradual culture change regarding reproducibility and openness of health-related data and results and the valuing and demanding of this culture by scientific journals (which is already happening today), coupled with the provision of additional means of technical and methodological support [e.g., repositories for long-term preservation of data such as (82) or repositories of community-contributed protocols used in data acquisition process such as (83)].

Another, but probably limited, solution is to rely on artificial intelligence methods that can extract some relevant information even from large amounts of raw, noisy data.

## Footnotes

^1^Available at: https://researchops.community.

^2^Available at: https://researchops.community/resources (Accessed 2022-06-08).

^3^Available at: https://www.go-fair.org/resources/internet-fair-data-services/ (Accessed 2022-06-08).

^4^Available at: https://ec.europa.eu/research/openscience/index.cfm?pg=open-science-cloud (Accessed 2022-06-08).

^5^Available at: https://bioportal.bioontology.org/ (Accessed 2022-06-08).

^6^Available at: http://ontofox.hegroup.org/ (Accessed 2022-06-08).

^7^Available at: http://www.ontobee.org/ (Accessed 2022-06-08).

^8^Available at: https://neuinfo.org/ (Accessed 2022-06-08).

^9^Available at: https://www.ebi.ac.uk/ols/index (Accessed on 2022-06-08).

^10^The NCIT ontology is available at https://bioportal.bioontology.org/ontologies/NCIT?p=summary (Accessed 2022-06-08).

^11^The NEMO ontology is available at http://bioportal.bioontology.org/ontologies/NEMO (Accessed 2022-06-08).

^12^More information is available at https://github.com/G-Node/nix/wiki/Model-Definition (Accessed 2022-06-08).

^13^More information about the odML terminologies can be found at https://github.com/G-Node/odml-terminologies (Accessed 2022-06-08).

^14^More information can be found at https://g-node.github.io/python-odml/ (Accessed 2022-06-08).

^15^The BiN ontology can be found here: https://bioportal.bioontology.org/ontologies/BIN/?p=summary (Accessed 2022-06-08).

^16^The BiN ontology can be found here: https://bioportal.bioontology.org/ontologies/BIN/?p=summary (Accessed 2022-06-08).
